# Evolutionary reframing – the case of melatonin

**DOI:** 10.1007/s00709-026-02227-5

**Published:** 2026-06-08

**Authors:** Peter Nick

**Affiliations:** Karlsruhe Institute of Technology, Joseph Gottlieb Kölreuter Institute für Plant Sciences, Karlsruhe, Germany

The origin of innovation has always been one of the harder nuts to crack in evolution biology. To explain how several genes simultaneously undergo compatible changes enabling a new function is difficult, because a selective advantage of these changes would be expected to result only from the accomplished final stage, not from its precursors that most likely would be dysfunctional. This Achilles’ Heel of evolution theory is often used by proponents of Intelligent Design under the term ‘Irreducible Complexity’ (Behe 2002) to undermine confidence into the fact of evolution as such. Often, years of hard research are needed to reach the level of knowledge required to come up with explanations to show that the complexity is, indeed, reducible and can result from a continuous evolutionary process (see, for instance, using the example of flagellar rotation in bacteria, Clements et al. 2009; in debunking Behe 2002). A common theme of evolutionary innovation is the recruitment of organs, processes, or proteins for a new function rather than “designing” them from scratch. This “evolution by tinkering” (*in sensu* Jacob, 1977) enables adaptation of these organs, processes, or proteins to selective pressures already before they face their new function. Often a peripheral or even “neutral” (in terms of fitness) consequence of this “pre-adaptation” can, if shifted to a new functional context, confer a selective advantage. To avoid the teleological connotations of the term “pre-adaptation”, Gould and Vrba (1982) proposed the term exaptation. While the phenomenon has been demonstrated in numerous cases, this term was not really generally adopted. Nevertheless, two contributions to the current issue can serve as illustration for such an exaptation, bridging animals and plants:

Melatonin, progressively popular as ailment against imsomnia, accumulates in the pineal gland following a circadian rhythm peaking in the night. For the synthesis of melatonin, its precursor serotonine needs to be acetylated by the key enzyme arylalkylamine-N-acetyltransferase, whose activity is negatively regulated by light perceived in the retina releasing a signal conveyed to the suprachiasmatic nucleus of the hypothalamus and from there further on to the pineal gland (for review see Tordjman et al. 2017). Due to this highly specific and complex context, melatonin would appear as an achievement of higher vertebrates at first sight. It came as a surprise, therefore, when it was discovered across many eukaryotes, even in the dinoflagellate *Gonyaulax polyedra*, where the rhythmicity of this hormone controls the daylength response of encystment (Hardeland et al. 1995).

The review by Hussain et al. (2026), in the current issue, explores the primordial functions of melatonin in plants. After its discovery in the 1990ies (due to its accumulation under adverse conditions including drought and salinity), it took some time until melanin became accepted as phytohormone. A review of melatonin responses to different abiotic and biotic stresses support the idea that this response is linked with its antioxidative effects. Typical contents are in the range of 1 ng per g freshweight, which is considerable comparing to the 10–30 pg per mL found in the blood plasma. The authors continue with signalling, starting with a short discussion of molecular candidates for a melatonin receptor and then systematically describing the interaction of melatonin with the major phytohormones, giving numerous examples that are also of applied relevance. For instance, in rice challenged by fluoride toxicity (a common problem in India and Bangladesh, where contaminated ground water is used for irrigation), melatonin can promote gibberellin synthesis and, thus, help to sustain plant growth under these adverse conditions (Singh et al. 2022). The authors finish with a short survey of current topics and research gaps in melatonin research especially perception and early signalling, and advocate more standardised protocols for melatonin extraction and detection for the sake of better comparability across different applications and crop species.

The work by Silva et al. (2026) can serve as a specific case study illustrating the role of melatonin for stress-related metabolism. Brazilian Ginseng (*Pfaffia glomerata*), a member of the Amaranthaceae, is used in indigenous medicine to cure diabetes and chronic inflammations. The medicinal effect is linked with the phytosteroid 20-hydroxyecdyson that accumulates in the root and can be boosted by salt stress (Fortini et al. 2023). In the current work, authors addressed the question, how this valuable plant can cope with osmotic stress, simulating drought episodes as they become more frequent due to climate change. As expected, they observed oxidative stress, growth inhibition, and chlorophyll loss. Application of melatonin as powerful antioxidant was, in fact, restoring root growth under osmotic stress, but was not effective in the shoot, although it increased the activity of superoxide dismutase, scavenging the highly reactive superoxide into hydrogen peroxide, and peroxidases, enzymes that can dissipate hydrogen peroxide. Furthermore, melatonin promoted the osmoprotective accumulation of sucrose and proline. However, accumulation of 20-hydroxyecdyson was evoked by osmotic stress, while melatonin was rather suppressive to this phytosteroid. Thus, while exogenous melatonin can help to mitigate drought stress, the data do not provide any evidence for a role of endogenous melatonin as osmoprotective hormone.

The link between the (primordial) function of melatonin as powerful antioxidant and its (derived) function as dark sensor provides an impressive example of exaption *in sensu* Gould and Vrba (1982). Light instability of melatonine is not a property of melatonin *per se*, but is strongly promoted under oxidative stress, mainly by the activity of superoxide in presence of a protoporphyrine moiety (Hardeland et al. 1995). Under these conditions, melatonin is easily oxidised to the breakdown product N^1^-acetyl-N^2^-formyl-5-methoxykynuramine (AFMK). These light-dependent antioxidant properties might have pre-disposed melatonin as sensor of light-dark cycles. Its well-known function as the “hormone of darkness” might, therefore, be an exaptation of its original function as powerful antioxidant buffering oxidative stress in the context of protoporphyrines, a function that is central for the stress resilience of organisms that use chlorophyll. Overall, innovation did not derive from new molecules, but from new functional contexts.
